# Ectopic Transplastomic Expression of a Synthetic MatK Gene Leads to Cotyledon-Specific Leaf Variegation

**DOI:** 10.3389/fpls.2018.01453

**Published:** 2018-10-04

**Authors:** Yujiao Qu, Julia Legen, Jürgen Arndt, Stephanie Henkel, Galina Hoppe, Christopher Thieme, Giovanna Ranzini, Jose M. Muino, Andreas Weihe, Uwe Ohler, Gert Weber, Oren Ostersetzer, Christian Schmitz-Linneweber

**Affiliations:** ^1^Institut für Biologie, Humboldt-Universität zu Berlin, Berlin, Germany; ^2^Computational Regulatory Genomics, Berlin Institute for Medical Systems Biology, Max Delbrück Center for Molecular Medicine, Berlin, Germany; ^3^Helmholtz-Zentrum Berlin für Materialien und Energie, Joint Research Group Macromolecular Crystallography, Berlin, Germany; ^4^Department of Plant and Environmental Sciences, The Alexander Silberman Institute of Life Sciences, The Hebrew University of Jerusalem, Jerusalem, Israel

**Keywords:** chloroplast, variegation, MatK, splicing, transplastomic, *Nicotiana tabacum*

## Abstract

Chloroplasts (and other plastids) harbor their own genetic material, with a bacterial-like gene-expression systems. Chloroplast RNA metabolism is complex and is predominantly mediated by nuclear-encoded RNA-binding proteins. In addition to these nuclear factors, the chloroplast-encoded intron maturase MatK has been suggested to perform as a splicing factor for a subset of chloroplast introns. MatK is essential for plant cell survival in tobacco, and thus null mutants have not yet been isolated. We therefore attempted to over-express MatK from a neutral site in the chloroplast, placing it under the control of a theophylline-inducible riboswitch. This ectopic insertion of MatK lead to a variegated cotyledons phenotype. The addition of the inducer theophylline exacerbated the phenotype in a concentration-dependent manner. The extent of variegation was further modulated by light, sucrose and spectinomycin, suggesting that the function of MatK is intertwined with photosynthesis and plastid translation. Inhibiting translation in the transplastomic lines has a profound effect on the accumulation of several chloroplast mRNAs, including the accumulation of an RNA antisense to *rpl33*, a gene coding for an essential chloroplast ribosomal protein. Our study further supports the idea that MatK expression needs to be tightly regulated to prevent detrimental effects and establishes another link between leaf variegation and chloroplast translation.

## Introduction

Land plant chloroplast RNAs are heavily processed post-transcriptionally. Next to trimming, and RNA editing, a prominent post-transcriptional processing step is RNA splicing of group II introns. Group II intron splicing is mediated predominantly by nuclear-encoded RNA binding proteins, of which about a dozen have been described in maize or Arabidopsis (de Longevialle et al., 2010; Stern et al., 2010) In addition to this nuclear complement of splicing factors, there is a single chloroplast-encoded splicing factor named intron maturase K, short MatK. The MatK protein is related to prokaryotic intron maturases (Neuhaus and Link, 1987). Bacterial intron maturases are encoded within group II introns, i.e., their “home introns.” The home intron of MatK is located in the *trnK* gene.

Group II introns are characterized by six secondary structure elements named domain (D) I – VI that fold into a globular tertiary structure (Michel and Ferat, 1995). The maturase reading frame is always found inside DIV, and the maturases are typically required for splicing of their host intron RNAs (Schmitz-Linneweber et al., 2015). Maturases are generally identified by several conserved motifs. These include a region with sequence similarity to retroviral-type reverse transcriptases (i.e., the RT domain), and a conserved sequence motif similar to the thumb domain of retroviral RTs, denoted domain X (Mohr et al., 1993). Biochemical studies have indicated that maturases make direct contacts with selected intron sequence elements and these contacts are essential for the splicing reaction. For example, the bacterial Maturase LtrA interacts with sequence stretches within DI, DII and DIV. These contacts help to attain a splicing-competent intron conformation (Matsuura et al., 2001; Rambo and Doudna, 2004). A major advance in the understanding of the roles of maturases in splicing has been recently accomplished by structural analyses of bacterial MATs bound to their cognate group II intron RNA targets (Piccirilli and Staley, 2016; Qu et al., 2016; Zhao and Pyle, 2016). These including the crystal structures of the RT domains of MATs from *Roseburia intestinalis* and *Eubacterium rectale* (Zhao and Pyle, 2016), and a cryo-EM analysis of the ribonucleoprotein complex of the *Lactobacillus lactis* intron-encoded LtrA maturase bound to its host *ltrB* intron RNA (Qu et al., 2016). The structures of the spliced *ltrB* intron (at 4.5 Å resolution) and of the *ltrB* intron in its ribonucleoprotein complex with LtrA (at 3.8 Å resolution) are further revealing functional coordination between the intron RNA with its cognate maturase protein. Remarkably, these structures reveal close relationships between the RT catalytic domain and telomerases, whereas the ‘active splicing centers’ resemble that of the Prp8 protein (Dlakic and Mushegian, 2011; Galej et al., 2013; Yan et al., 2015), which also resides at the core of the spliceosome.

Aside of their essential role in splicing, intron maturases are also required for genetic mobility of the intron (Lambowitz and Zimmerly, 2004). Bacterial group II introns can spread to other genomic positions in a process called retrohoming that depends on the maturase protein. Chloroplast group II introns of embryophytes are no longer mobile and MatK has lost the protein domains required for intron mobility (Mohr et al., 1993; Barthet and Hilu, 2007). By contrast, it has retained the so-called domain X, which was shown to be required for RNA splicing activity of bacterial introns (Lambowitz and Zimmerly, 2004).

A role in splicing for MatK is strongly supported by studies on chloroplasts devoid of a translational apparatus. In these mutants, the *trnK* precursor-RNA is not spliced (Vogel et al., 1997). In addition, an entire subgroup of chloroplast introns termed group IIA introns fails to splice as well (Hess et al., 1994; Vogel et al., 1999). The only conceivable factor that would require functioning chloroplast translation for splicing is MatK, which led to the proposition that MatK serves splicing of all group IIA introns. Indeed, direct association of MatK with intron RNA was demonstrated *in vitro* (Liere and Link, 1995) and with seven group IIA introns also *in vivo* (Zoschke et al., 2010).

The *matK* reading frame is found in all known autotrophic land-plant chloroplast genomes that contain group II introns, and is also present in basal streptophyte algae (Turmel et al., 2006). In the streptophyte algae *Zygnema*, in the fern *Adiantum capillus-veneris* and also in the parasitic land plants *Epifagus virginiana, Cuscuta exaltata*, and *Cuscuta reflexa, matK* is present as a stand-alone reading frame, while the *trnK* gene has been lost (Wolfe et al., 1992; Turmel et al., 2005; Funk et al., 2007; McNeal et al., 2007). This suggests a function of *matK “in trans,”* most likely for the splicing of pre-RNAs other than its cognate *trnK* intron. Among all embryophytes analyzed, only parasitic species have lost *matK*, among them species in the genera *Cuscuta, Cytinus, Rafflesia, Pilostyles*, as well as the orchid *Rhizantella* and other orchid genera (Funk et al., 2007; McNeal et al., 2009; Delannoy et al., 2011; Molina et al., 2014; Bellot and Renner, 2015; Roquet et al., 2016). These non-autotrophic plants have also lost their group IIA intron sequences, with the exception of the structurally derived and ‘evolutionarily younger’ *clpP*-2 intron (Turmel et al., 2006; Funk et al., 2007; McNeal et al., 2009). Together, these phylogenetic analyses further support the notion that MatK is required for more than just splicing of its home intron. Also, its continuous presence in chloroplast genomes starting from early streptophytes suggests that its retention inside the chloroplast is not a chance event. Unlike *matK*, many other chloroplast and also mitochondrial genes have been transferred multiple times in different land plant lineages to the nucleus (Kleine et al., 2009). This includes several genes coding for maturases that have been found in the Arabidopsis nuclear genome (Mohr and Lambowitz, 2003). These are all targeted to the mitochondria (one is dually targeted to the mitochondria and chloroplasts), and at least three of them serve splicing of multiple mitochondrial group II introns (Nakagawa and Sakurai, 2006; Keren et al., 2009, 2012; Cohen et al., 2014; Zmudjak et al., 2017). These data demonstrate that maturases can be transferred to the nucleus and still function in splicing *in trans*. An important question therefore is: ‘why is *matK* evolutionary maintained on the chloroplast chromosome’?. To address this, functional genetic analyses are required. Unfortunately, the *matK* gene has been recalcitrant to reverse genetic tampering. Mutagenesis of the chloroplast *matK* reading frame by transplastomic mutagenesis failed so far, which has been taken as evidence that *matK* is an essential gene (Drescher, 2003; Zoschke et al., 2010). We therefore decided to study *matK* using a gain of function approach, i.e., by ectopic over-expression from the chloroplast genome in addition to the endogenous copy.

## Results

### Introducing an Additional Copy of Chloroplast MatK Into a Neutral Integration Site Leads to Homoplastomic Tobacco Lines With Variegated Cotyledons

**FIGURE 1:**
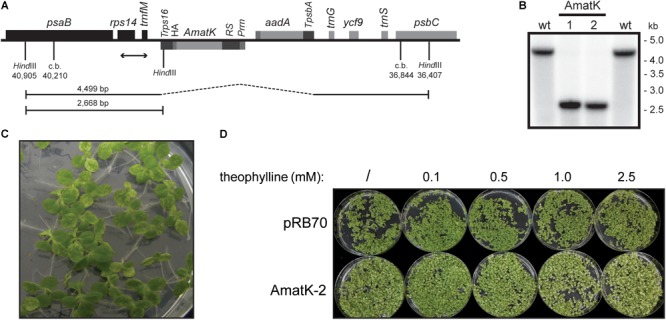
Generation of transplastomic tobacco plants with a riboswitch-induced synthetic *matK* gene. **(A)** Schematic map of the transformed plastome containing the *aadA* cassette and a riboswitch-driven cassette including the *matK* gene (AmatK). c.b. = construct border. Numbers refer to genome position within the tobacco chloroplast genome (accession Z00044.2). **(B)** Southern blot analysis to test for integration of the transgenic cassettes. Total DNA was digested with the restriction enzyme *Hind*III, which generates fragments of 4499 and 2668 bp as indicated by handlebars in **(A)**. The position of the DNA probe used is shown by a double-headed arrow in **(A)**. **(C)** Phenotype of 12 day old seedlings grown *in vitro*. **(D)** Seed germination of control plants and AmatK plants on media containing different amounts of theophylline. Plants were photographed after 10 days of growth.

Given that *matK* is considered to be an essential gene, we opted for engineering an inducible over-expressor of MatK to avoid lethal affects after constitutive over-expression. Therefore, we used a transformation vector that allows the expression of transgenes based on the theophylline-responsive riboswitch (Verhounig et al., 2010). In brief, the riboswitch utilized in this system is a translational on-switch, i.e., modulates translation initiation through the formation of an alternative structure that sequesters the Shine-Dalgarno and initiation (AUG) codon sequences in the absence of the inducer. The addition of theophylline leads to conformational changes in the 5′-UTR, which serves as an entry point for chloroplast ribosomes, allowing the translation of the transgene (Winkler and Breaker, 2005). This system was successfully used for the induced expression of GFP, using the chloroplast transformation vector pAV (Verhounig et al., 2010). The pAV vector also contains a selectable marker, i.e., a chimeric spectinomycin resistance gene, *aadA*, that facilitates the selection of plants with transgenic chloroplast genomes. The vector includes flanking sequences that allow to target the transgenes to a neutral intergenic spacer region in the chloroplast genome of tobacco by homologous recombination. Here, we replaced the GFP construct within the pAV vector with a *matK* reading frame, that has only 72% nucleotide sequence similarity with the endogenous tobacco *matK* sequence, while maintaining the encoded amino acid sequence (resulting vector: pRSAmatK, see also **Figure 1A** and **Supplementary Figure S1**). This prevents undesirable homologous recombination events within the endogenous *matK* sequence. In addition, we added a C-terminal triple HA tag to allow detection of the transplastomic MatK protein. Previously, we showed that C-terminal tagging of the endogenous MatK does not interfere with intron splicing and does not entail any detectable macroscopic phenotype (Zoschke et al., 2010).

The pRSAmatK construct was introduced into tobacco plants by biolistic transformation of the chloroplast genome, followed by selection of spectinomycin-resistant cell lines. We isolated a total of 11 putative transplastomic lines. All lines were subjected to additional rounds of regeneration under antibiotic selection. This eliminated residual wild-type copies of the plastid genome, i.e., produced homoplastomic lines. We further tested for homoplastomy and correct integration by restriction fragment length polymorphism analyses (**Figure 1B**). We concluded that the lines were homoplastomic. We named these lines AmatK to reflect the use of a synthetic *matK* reading frame. When growing F1 plants from various AmatK lines, we noticed the mottled, variegated appearance of cotyledons in AmatK seedlings (**Figure 1C**). By contrast, the primary leaves were indistinguishable from those of the wild-type plants (**Figure 1C** and **Supplementary Figure S2**; in some cases, pale-green tissue was also observed in early primary leaves). The effect on the plants in the absence of the riboswitch inducer, theophylline, can be attributed to the known leakiness of the construct (Emadpour et al., 2015). We next asked, whether addition of theophylline to AmatK lines would modulate the observed variegated phenotype. Indeed, increasing concentrations of theophylline in the growth medium exacerbated cotyledon bleaching, while the control plants did not respond visibly to the addition of theophylline (**Figure 1D**). Together, these phenotypic analyses indicate that the *AmatK* insertion (i) interferes with chloroplast biogenesis in a cell-autonomous fashion, (ii) is restricted to cotyledon tissue, (iii) and responds to the riboswitch activation.

### *AmatK* Leaf Variegation Is Modulated by Light, Sugar and the Inhibition of Chloroplast Translation

**FIGURE 2:**
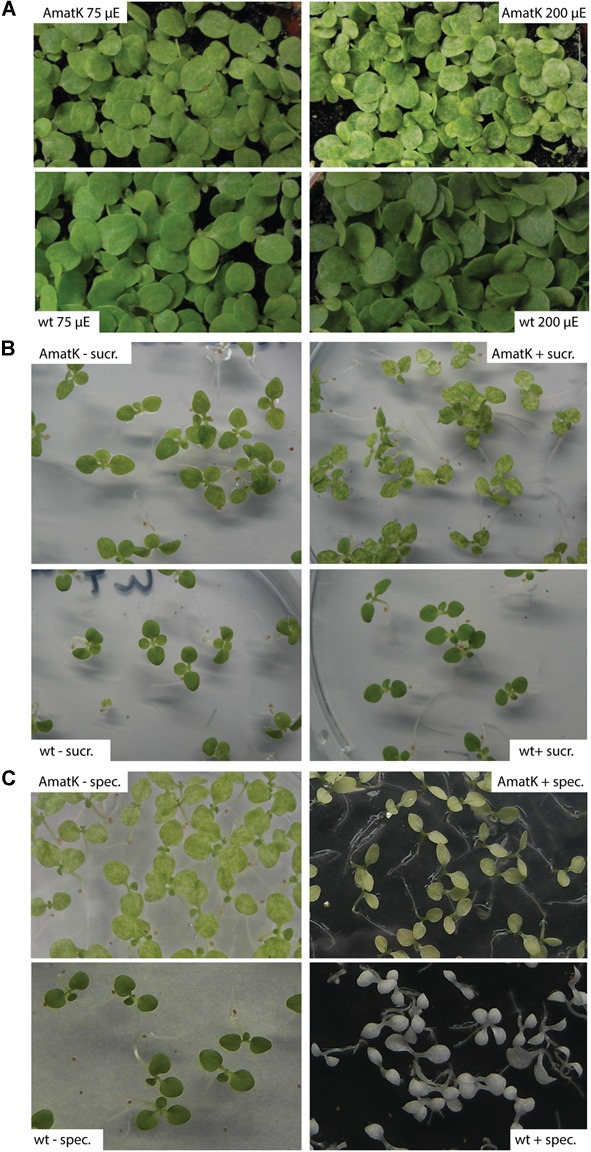
AmatK variegation is modified by light, sugar and translation inhibition. **(A)** AmatK and wt plants germinated under two light regimes and photographed after 10 days. **(B)** AmatK and wt plants germinated on MS media with or without a sucrose supplement (3%w/v). **(C)** AmatK and wt plants germinated on MS media with or without a spectinomycin supplement (500 μg/L).

Variegation of genetically homogenous tissue has been observed before, and often was shown to be modulated by external signals. For example, variegation of the *immutans* mutant is strongly affected by light and temperature signals (Rodermel, 2002). We noticed that variegation was variable depending on in which growth cabinet and under which conditions we grew the plants. Both, the extent of pale areas and different degrees of paleness were observed. We therefore systematically tested the effects of different growth conditions on the extent of variegation. Specifically, we applied conditions that are known to affect photosynthesis – the core function of chloroplast genetic information. First, we tested different light conditions. At higher light fluences of 200 μE m^-2^⋅s^-1^, AmatK cotyledons are paler than at 75 μE m^-2^⋅s^-1^, while wt plants tolerate both light conditions with no visible alteration in leaf coloration (**Figure 2A**). This indicates that the ectopic insertion of *AmatK* leads to sensitivity to higher light intensities, i.e., potentially to a compromised photosynthetic apparatus and thus eventually to photoinhibition. Noteworthy, plants grown on soil show less severe variegation and paleness than plants grown *in vitro* on MS medium. This indicates that different growth conditions, like for example the provision with nutrients, gas exchange and/or light differences between the petri-dish grown plants and the soil-grown plants affect variegation as well.

Sugar is known to be a key regulator of plant metabolism, affecting the expression of many different plant genes (Pego et al., 2000). Accordingly, photosynthesis is known to be affected by the availability of exogenous sugars, which are thought to limit photosynthesis and prevent the proper development of the photosynthetic apparatus (Hdider and Desjardins, 1994; Koch, 1996; Van Huylenbroeck and Debergh, 1996; Rybczyński et al., 2007). AmatK plants grown on sugar show paler cotyledons, with larger patches of reduced chlorophyll, while wt plants were unaffected, suggesting that the effects of sugar and the AmatK transgene are additive (**Figure 2B**).

MatK has been suggested to be important for splicing of RNAs with essential roles for the translational apparatus, including several tRNAs and mRNAs for ribosomal proteins. Therefore, the effect of *AmatK* might well run via partially compromised translational activity in plastids. This should in turn effect the expression of the selectable marker introduced into AmatK plants, the *aadA* cassette. *aadA* confers resistance against spectinomycin. We tested spectinomycin resistance by germinating AmatK seeds on spectinomycin containing medium. As expected, wt seedlings are albino, devoid of chloroplast development. AmatK lines show yellowish cotyledons with almost no green sectors remaining (**Figure 2C**). We conclude that an inhibition of translation exacerbates variegation of AmatK cotyledons.

### Transgene-Derived mRNA, but No Full-Length MatK:HA Protein Is Detected in AmatK Lines

By design, the *AmatK* transgene was supposed to be translationally silent in the absence of the inducer theophylline. Yet, even in the absence of theophylline, AmatK plants showed a variegated cotyledon phenotype. These effects may be due to basal expression in the absence of inducer (Emadpour et al., 2015). Possibly, low expression of *AmatK* already impacts chloroplast development at least in cotyledons. We therefore tested expression of the *AmatK* gene on the RNA and protein level.

**FIGURE 3:**
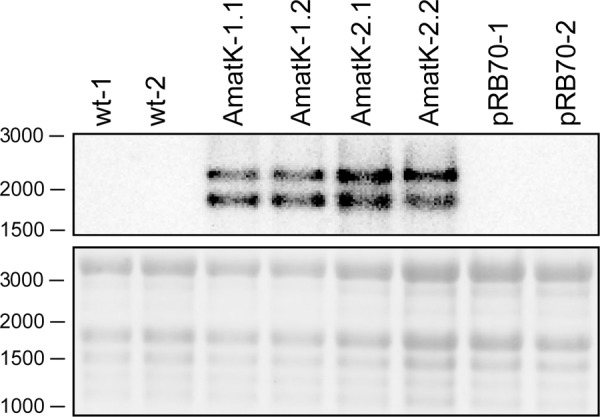
Detection of synthetic *matK* transcripts from AmatK, wt and *aadA* leaf tissue using RNA gel blot hybridization. 5 μg of total plant RNA was extracted and analyzed by RNA gel blot hybridization using a probe directed against the junction of the AmatK and HA sequence, which is specific to the transgene. The wt lines -1 and -2 as well as the AmatK lines -1 and -2 represent biological replicates.

RNA gel blot analyses were carried out with total RNA from AmatK lines grown on MS-medium with 75 μE m^-2^⋅s^-1^ of light at 25°C (16 h light, 8 h darkness). Plants were harvested 12 days after imbibition. The oligonucleotide used for probe preparation is complementary to the junction region of the *AmatK* reading frame and the triple HA-tag, ensuring that the signal obtained from radioactive probe hybridization is specific to transcripts of the *AmatK* sequence. Two biological replicates were analyzed for each of two *AmatK* lines. In addition, we extracted RNA from two independent wt samples and two plants from the previously described pRB70 line, which carries an *aadA* cassette without the additional *AmatK* gene within the intergenic region between *trnfM* and *trnG* (Ruf et al., 2001). This controls for unwanted signals from the strongly expressed selectable marker gene. The *AmatK* lines display two signals at 1.8 and 2.2 kb, while no signal was observed for wt or pRB70 control samples (**Figure 3**). The expected size for a complete transcript of the synthetic *matK* gene spanning the region from the Prrn promoter to the *rps16* terminator sequence is approximately 1.8 kb. Termination of transcription in chloroplasts is poorly understood, and transcriptional read through across so-called terminators of transcription of transplastomes has been seen before (Schmitz-Linneweber et al., 2001). Therefore, the longer, 2.2 kb transcript possibly represents read-through transcription across the *rps16* terminator and ends at the downstream *trnfM* gene. The absence of both signals from wt RNA samples demonstrates that the signals are not due to endogenous RNA species but represent mRNAs derived specifically from the introduced transgene.

We next tested accumulation of the transgene-derived MatK protein using an antibody against the C-terminal HA epitope. We prepared total plant protein and/or stroma from AmatK and wt lines. Some AmatK plants were grown in the presence of the riboswitch inducer theophylline. We used the following controls: (1). As controls for potential signals from the *aadA* cassette, we used the PRB70 lines mentioned above. (2). To control for effects caused by the pale cotyledon tissue, we used a phenocopy generated by treating wt plants with low doses of spectinomycin. This treatment leads to bleached cotyledons, but still allows greening of primary leafs, i.e., mimics the phenotype of AmatK seedlings (**Supplementary Figure S3**). (3). As a positive control, we added a line that carries an HA-tagged plastome-encoded subunit of the plastid RNA polymerase named RpoA:HA (Finster et al., 2013). (4). Total protein preparations from plants that carry an N- or C- terminally HA-tagged MatK, designated HA:MatK or MatK:HA, (Zoschke et al., 2010), were used as positive controls for the detection on MatK in transformed tobacco plants.

**FIGURE 4:**
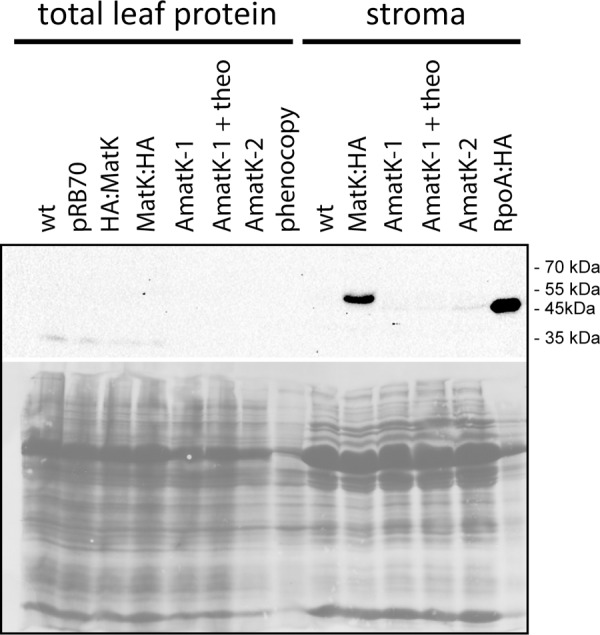
Immunological detection of MatK:HA in AmatK lines and various controls. Top: Total leaf proteins were denatured and separated on a 12% polyacrylamid gel, blotted and probed with a rabbit anti-HA antibody. pRB70 = control lines containing an *aadA*-cassette, but no riboswitch cassette; HA:MatK = line with an N-terminal HA-tagged endogenous *matK* gene. MatK:HA = line with a C-terminal HA-tagged *matK* gene; theo = theophylline treated AmatK plant; phenocopy = wt plants treated with 17 mg/L spectinomycin; RpoA:HA = line with a C-terminally HA-tagged *rpoA* gene. Bottom: ponceau S stain of the blot shown above.

Our immunoblots show that MatK can be detected in stroma preparations from the tagged endogenously locus, confirming previous analyses (Zoschke et al., 2010) (**Figure 4**). The protein runs at approximately 55 kDa, well below the calculated molecular weight of 64 kDa, but in line with previous analyses of MatK (Zoschke et al., 2010). We could not detect MatK in any sample from total leaf protein preparations. Also, no signal corresponding to the size of the MatK:HA protein was observed in AmatK lines, including lines treated with theophylline, and independent of sample preparation (stroma or total protein). However, all stroma preparations from AmatK lines had a faint signal of about 47 kDa, possibly a degradation product (**Figure 4**). This signal is specific to the AmatK lines and is found consistently in different probings (**Supplementary Figure S4**). The signal must represent a C-terminal fragment of MatK, since it is detected by a C-terminal tag. In sum, our analysis indicates that protein accumulation from the synthetic transgene is aberrant and thus does not represent an over-expressor. While the transgene is expressed on the RNA level, no full-length MatK was detected on the protein level. Still, AmatK leads to the accumulation of a well-defined N-terminally truncated MatK fragment and a tissue specific chloroplast defect. This variegated tissue was used for all further experiments, without further transgene induction by theophylline.

### Chloroplast RNA Splicing Is Unaffected in AmatK Lines

**FIGURE 5:**
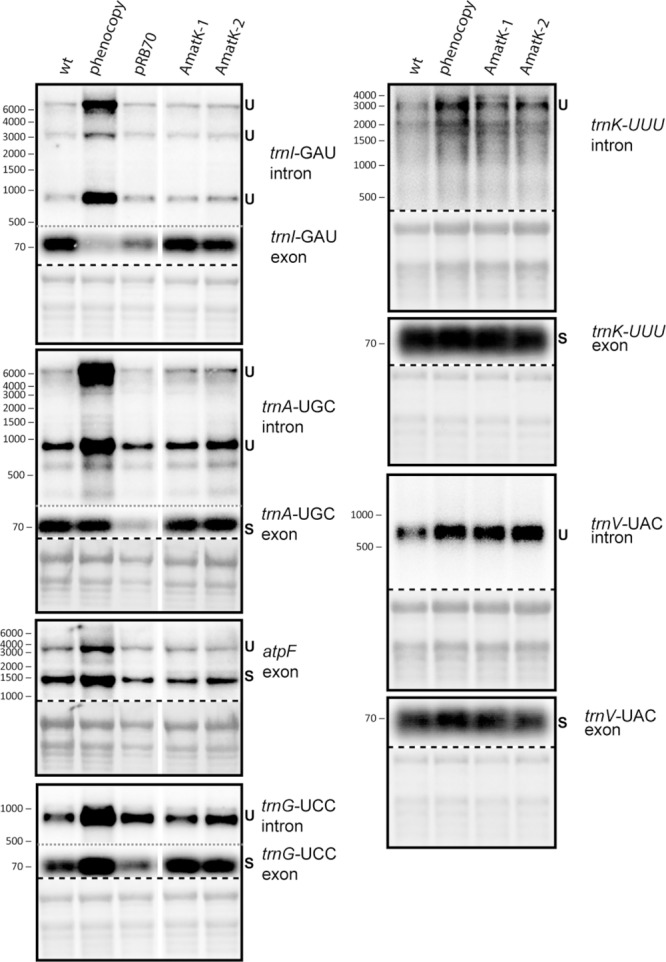
Analysis of RNA splicing in AmatK and control plants. 5 μg of total leaf RNA was separated on 1.2% agarose gels and analyzed by hybridization to probes specifying target RNAs of MatK (see **Supplementary Table S1** online for primer sequences). The following RNA samples were analyzed: RNA from AmatK, *aadA*, wt plants, and wt plants trested with spectinomycin (phenocpy control). Methylene blue staining identifies rRNAs and was carried out prior to hybridization. The autoradiographs and the corresponding methylene blue stains (below) are separated by a dashed line. Note that exon probings for *trnG, trnA*, and *trnI* were done after the respective intron probings on the same blots (separated by gray, dotted lines). White vertical lines indicate, where irrelevant lanes have been removed.

There is as yet no formal proof, but strong biochemical and phylogenetic evidence for MatK being a splicing factor. Splicing defects could explain the altered chloroplast development in AmatK, in particular since MatK associates with four tRNA genes (Zoschke et al., 2010), which are essential for translation and thus chloroplast development (Alkatib et al., 2012a,b). We therefore analyzed total RNA from young seedlings at the cotyledon stage for the accumulation of spliced and unspliced RNAs that are considered MatK targets based on previous RNA-co-immunoprecipitation assays (Zoschke et al., 2010). We include phenocopies generated by mild spectinomycin treatment to control for secondary effects caused by failed chloroplast development. Both, probes complementary to intron as well as exon sequences were used to detect spliced as well as unspliced RNAs. Accumulation of unspliced versus spliced RNAs would be indicative of a splicing effect. However, no such differential accumulation was observed in AmatK lines versus controls (**Figure 5**). The stronger signals for unspliced precursors seen for *trnK* and *trnV* in AmatK samples versus wt samples are also seen for the phenocopy control and appear to be caused by differences in loading (see methylene blue stains). In sum, RNA splicing of the analyzed target RNAs is not impaired, arguing against an RNA splicing effect leading to the observed variegation.

### AmatK Plants Accumulate RNA Antisense to *rpl33* When Treated With Spectinomycin

**FIGURE 6:**
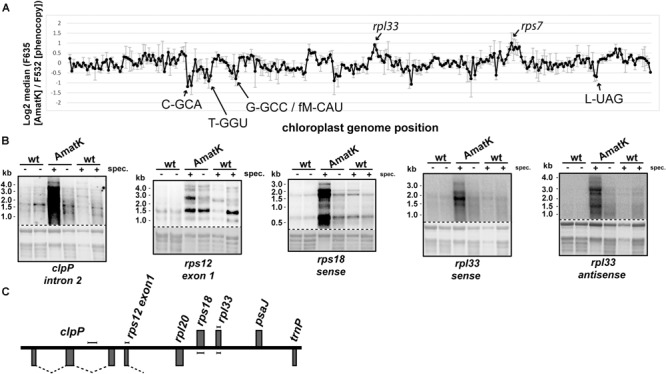
Expression analysis of the AmatK chloroplast genome. **(A)** Microarray survey experiment of chloroplast RNAs. Two μg of total leaf RNA from 10 day old AmatK plants and wt plants treated with low doses of spectinomycin (phenocopy control) were labeled with Cy5 (F635) and Cy3 (F532), respectively, and hybridized to a tobacco whole plastid genome tiling microarray. Each PCR probe was present in 12 replicates on the array and two biological replicates were performed. The average and standard deviation of the log2 signal ratios F635/F532 across the biological replicates was calculated and plotted agains the genomic position on the tobacco plastid genome (accession Z00044.2). Increase or decrease of RNA from AmatK plants relative to phenocopy controls was not significant (FDR > 0.05), rarely reaching a log2 fold-change of 2. We nevertheless labeled peak areas of enrichment. **(B)** RNA gel blot analysis of the *rpl33*-*clpP* genomic region. 5 μg of total plant RNA from AmatK and wt plants was separated on a 1.2% denaturing agarose gel and probed with strand specific RNA probes as indicated below each autoradiograph. Some plant samples were treated with spectinomycin (spec.). Loading and RNA integrity were controlled by staining rRNAs with methylene blue (below autoradiographs; The autoradiographs and the corresponding methylene blue stains are separated by a dashed line). **(C)** Schematic representation of the *clpP-rpl33* genomic region. Genes on top of the line are transcribed from right to left, genes below the line from left to right. Probes used for the RNA gel blot experiments shown in **(B)** are indicated by handle bars. Introns are indicated by dashed lines. Note that the intron in *rps12* is trans-spliced, i.e., only the first part of the intron is located within this genomic locus.

In the absence of specific splicing defects, we wanted to examine other parts of chloroplast RNA metabolism in AmatK lines. Therefore, we analyzed global chloroplast RNA accumulation by microarray analysis. To this end, we extracted RNA from AmatK seedlings as well as from spectinomycin-treated phenocopy controls. The AmatK and phenocopy control RNAs were labeled with Cy3 and Cy5 fluorescent dyes, respectively, and hybridized competitively on a microarray that represents the chloroplast genome of tobacco in a tiling fashion (Finster et al., 2013). However, none of the probes showed a significant change (**Figure 6A, Supplementary Table S1**, and **Supplementary Figure S5**). Mild reductions were seen for several tRNAs and a mild, but non-significant increased steady-state level was observed for two mRNAs (marked in **Figure 6A**). Of these, we randomly picked the *rpl33-clpP* region for validation by RNA gel blot hybridization. We probed four genes located on opposite strands, using strand-specific probes (**Figures 6B,C**). The steady state levels of these RNAs were found to be similar between wild-type and AmatK plants, further supporting the microarray data.

Given that spectinomycin exacerbates the bleaching phenotype (**Figure 2C**), we tested RNA accumulation also on spectinomycin-containing medium. Indeed, transcript levels are dramatically increasing for *rpl33, rps18* and *clpP* under these conditions (**Figure 6B**). An increase can also be observed for *rps12*, in particular when considering that less total RNA is loaded in this lane. Intended as a negative control, we also probed for RNA accumulating antisense to *rpl33*. To our surprise, we obtained strong signals for AmatK lines treated with spectinomycin, whereas for all other samples, signals were barely above background.

In sum, plastid RNA levels in AmatK plants are very similar to RNA levels in control plants, thus suggesting that the phenotype is not caused by altered RNA accumulation in the mutants. The situation changes drastically, when plants are treated with spectinomycin, which is also reflected in the more pronounced cotyledon bleaching: Spectinomycin induces over-accumulation of several mRNAs in AmatK lines and also leads to strongly increased antisense transcripts to the *rpl33* gene. In conclusion, inhibition of plastid translation in *AmatK* insertion lines leads to reprogramming of chloroplast gene expression at least for the *rpl33-clpP* genomic region.

## Discussion

### *AmatK*-Induced Plastome-Based Leaf Variegation Is Modulated by External and Internal Cues

The differentiation of chloroplasts in land plants depends foremost on light and developmental-dependent signals (Lopez-Juez and Pyke, 2005). A multitude of mutants perturbed in chloroplast development have been isolated by forward and reverse genetic approaches (Leister, 2003). Such mutants usually display uniform bleaching phenotypes, and are often embryonic lethal or seedling lethal. Some mutants have variegated leaves. Leaf variegation is based on the occurrence of tissue sectors that either contain fully developed chloroplasts or pale, aberrant plastids (Kirk and Tilney-Bassett, 1978; Sakamoto, 2003; Aluru et al., 2006). Recessive mutants that cause variegation have been isolated in monocot as well as in dicot plants (e.g., Hagemann and Scholz, 1962; Walbot and Coe, 1979; Carol et al., 1999; Wang et al., 2000, 2004). These are typically caused by nuclear-loci. By contrast, AmatK plants represent a chloroplast genetic alteration that is maternally inherited. Plastome-based variegation can be caused by segregation of two genetically distinct plastids, one of which does not develop into fully green chloroplasts (Baur, 1909; Correns, 1909; Börner and Sears, 1986; Greiner and Bock, 2013). It has also been described for the segregation of two genetically distinct plastid chromosomes, for example segregation of chromosomes lacking a *trnN-*GUU gene versus wt chromosomes (Legen et al., 2007). Depletion of *trnN*–GUU pools during segregation of homoplastomic tissue was considered to cause leaf variegation. This cannot be the case with AmatK plants, since these lines are homoplastomic according to Southern analyses. Thus, since AmatK plants are genetically uniform, variegation must be linked to a different, variable parameter. In AmatK cells, this parameter drops below a hypothetical threshold, which prevents chloroplast development. Three external factors, light, sugar and the antibiotic spectinomycin were shown to modulate the extent of variegation and are thus informative in terms of identifying the putative threshold. Light has been noted before as a booster of variegation (Rosso et al., 2009). Light intensity during growth correlates with pale leaf area in the variegation mutants *immutans, spotty, var1, var3*, and *thf1* (Rosso et al., 2009). This effect has been attributed to increased excitation pressure and the resulting photo-oxidative damage under higher light fluences (Rosso et al., 2009; Huang et al., 2013). In analogy, we hypothesize that the *AmatK* insertion makes developing chloroplasts more vulnerable to increased excitation pressure. Given the role of MatK as a splicing factor, altered gene expression in AmatK plants might interfere with the correct expression of the photosynthetic apparatus and thus lead to detrimental changes in electron transport that are exacerbated under high light conditions. The nature of the primary defect remains to be determined.

Sugar as the second modifier of the AmatK phenotype has been shown to lead to the repression of nuclear genes for photosynthetic functions, e.g., the light harvesting proteins (Krapp et al., 1991; Price et al., 2004; Li et al., 2006). In addition, sugar represses plastid gene expression in liquid culture (Price et al., 2004; Gonzali et al., 2006; Osuna et al., 2007) and also in seedlings grown on sugar-containing MS media (Van Dingenen et al., 2016). Interestingly, the effect on chloroplast gene expression was observed as early as 3 h following sucrose treatment – roughly half of all repressed genes are from the chloroplast genome (Van Dingenen et al., 2016). Notably, *matK* and *rps18* are the most strongly reacting non-photosynthetic chloroplast genes after sugar treatment (Table 1 in Van Dingenen et al., 2016). We hypothesize that the detrimental effect of AmatK on chloroplast gene expression adds up with the repressive effects of sugars on chloroplast gene expression (including on the endogenous *matK*), which ultimately results in increased variegation. This implies a threshold of plastid gene expression during chloroplast development, below which no differentiation of chloroplasts is possible. This is further supported by our results from spectinomycin treatments, which showed an even more pronounced effect on variegation than light and sugar application. Spectinomycin blocks chloroplast translation, but should be detoxified by the expression of the *aadA* cassette in AmatK plants. This function is compromised given the almost complete bleaching of cotyledons on spectinomycin-containing medium. In conclusion, the expression of the *aadA* cassette is hampered in the AmatK background. Expression of the *aadA* cassette is driven by a PEP promoter and requires an active and intact plastid translational machinery. In summary, our data suggest that the *AmatK* insertion negatively affects plastid gene expression, which in some cotelydon cells is lowered beyond a point necessary for chloroplast development.

### *AmatK* Is Involved in Cotyledon-Specific Chloroplast Development

The main difference between cotyledons and primary leaves is their developmental origin. While all primary leaves are derived from the shoot apical meristem (SAM) post-germination, cotyledons emerge already at the late globular stage of embryo development. This is a time in development, when seedlings are submerged in the soil and live heterotrophically. The plastids in cotyledons develop into etioplasts and only when they emerge from the soil and are irradiated, etioplasts transform into chloroplasts (Mansfield and Briarty, 1996). This is a very different trajectory than plastids take in the SAM, where they differentiate from proplastids directly into chloroplasts during leaf primordia development. The genetic basis of chloroplast development in cotyledons is mostly unknown, but a smaller number of mutants have been described that display pale or even albino cotyledons, while the primary leaves are able to undergo normal greening. This includes *white cotyledons* (*wco*; Yamamoto et al., 2000), and *snowy cotyledon 1, 2*, and *3* (*sco1,2,3*; Albrecht et al., 2006, 2008, 2010; Ruppel and Hangarter, 2007; Shimada et al., 2007; Zagari et al., 2017). Other mutants, including *sig6* and *dg1* mutants, exert a dominant effect on cotyledons, but also lead to measureable alterations in chlorophyll content in primary leaves (Ishizaki et al., 2005; Chi et al., 2008). Only one mutant has been described to date that leads to cotyledon-specific variegation, caused by a disruption of the *spd1* gene (Ruppel et al., 2011). All of the listed mutants are recessive and affect nuclear genes. *AmatK* is distinct in eliciting cotyledon variegation via a plastome modification. It is interesting that – like MatK – several of the mutated nuclear genes underlying variegation phenotypes are involved in plastid gene expression: *WCO* and *SCO1* have been implicated in rRNA maturation and translation elongation, respectively. SIG6 and DG1 interact with each other and play a role for plastid transcription. This demonstrates that defects in chloroplast gene expression can selectively affect chloroplast development in cotyledons – a possible scenario also for the AmatK lines. *SCO2* encodes a DNAJ-like protein and is required for the accumulation of photosystem II-LHCII complexes (Zagari et al., 2017). This mechanism for variegation is likely distinct from *AmatK*-induced variegation in that it does not involve plastid gene expression and since recent data indicate functions for SCO2 beyond cotyledons (Zagari et al., 2017). The functions of SPD1 and SCO3 are unknown. In sum, while the molecular mechanisms behind cotyledon-specific variegation remain unknown, it is intriguing that of the few factors known, most play a role for plastid gene expression. Thus, chloroplasts in cotyledons depend on a yet to be determined threshold of chloroplast gene expression, that is no longer crossed in all plastids during development in variegation mutants.

### A Gain of Function in AmatK Lines Impairs Chloroplast Development

The initial aim of our study was to generate a strong overexpressor of MatK. However, while *AmatK* mRNA is readily detected, we did not find any full-length MatK protein derived from the transgene, but instead only a weakly accumulating degradation product. Since the tag used for the detection of the degradation product is located at MatK’s C-terminus, we conclude that translation proceeds to the stop codon, and that the protein is degraded co- or post-translationally. This is surprising, since the amino acid sequence of AmatK is identical to the stable, endogenously HA-tagged version of MatK (Zoschke et al., 2010). We speculate that translation dynamics of the *AmatK* mRNA are different from those of the endogenous *matK* mRNA since the two genes have massively different codon usage (**Supplementary Figure S1**). Changes in translation speed can lead to different folding kinetics of the nascent protein and thus to alternative degradation routes (Kimchi-Sarfaty et al., 2007; Gloge et al., 2014). We speculate that such a truncated protein could act as a dominant negative factor in the chloroplast. However, the truncated AmatK protein levels are not rising after theophylline treatment, while at the same time variegation increases. Thus, the N-terminally truncated AmatK cannot be the cause of increased defective chloroplast development after theophylline addition. We, however, cannot rule out that an AmatK protein fragment representing the N-terminal part of AmatKs exerts the effect. Such a fragment would, however, not be detectable, because it would lack the C-terminal HA tag and thus remains a speculation at present. Alternative explanations, i.e., that the *AmatK* gene or the *AmatK* mRNA cause the defect, cannot be ruled out, but are also not in line with the theophylline-dependence of the phenotype. While this needs to be tested in the future, our data suggest that the downstream effects represent a gain of function rather than a loss of function of MatK. First, splicing of MatK target introns appears unaffected in AmatK plants: If we agree to take MatK as a splicing factor (Zoschke et al., 2010), these data suggest that AmatK does not compete or interfere with the endogenous MatK. Second, we discovered a number of effects on the accumulation of RNAs that are dependent on inhibiting translation in AmatK plants. These RNAs are not associated with MatK in RIP-chip assays. For instance, the *rpl33* operon accumulates more RNA in AmatK plants than in phenocopy controls, including the accumulation of antisense RNA. In the green alga *Chlamydomonas reinhardtii*, chloroplast antisense RNAs are stabilized in the absence of translation by forming double-stranded RNA (Cavaiuolo et al., 2017). In general, over-accumulation of RNAs has been described for mutants with global defects in chloroplast translation and is caused at least in part by an increased activity of selected chloroplast RNA polymerases in albino tissue (Emanuel et al., 2004). We tried to control for such secondary effects by analyzing phenocopy controls, but in fact cannot be sure, whether the extent of translational inhibition in the control is truly mimicking the effect seen in AmatK plants. Therefore, whether the RNA defects seen in AmatK tissue are primary or secondary effects cannot be decided at present. Still, a defect in the expression of *rpl33* and its neighboring genes, *rps18* and *rpl20*, could very well explain the bleaching phenotype. *rps18* and *rpl20* encode essential ribosomal proteins (Rogalski et al., 2006; Rogalski et al., 2008). The antisense RNA detected here is long enough to also make hybrids with *rps18* and *rpl20* mRNAs. This could interfere with sense translation of both mRNAs and thus eventually to reduced ribosome biogenesis and translation capacity. That reduced translational capacity can lead to bleached tissue is exemplified by spectinomycin treatment and several mutants in genes for translational components (e.g., Bellaoui and Gruissem, 2004; Legen et al., 2007; Meurer et al., 2017). Future transplastomic expression of antisense RNA to selected mRNAs could test this hypothesis. Importantly, the option to modulate the level of variegation in cotelydons of AmatK lines makes these plants a valuable tool to determine the time-window and unravel the threshold at which chloroplast gene expression exerts its effect on plastid differentiation.

## Materials and Methods

### Plant Material and Growth Conditions

Tobacco plants (*N. tabacum* cv. *Petit Havana*) were grown on agar medium containing 30 g/L sucrose for plastid transformation assays. Transplastomic AmatK or pRB70 plants were grown on the same medium or on medium supplemented with 500 mg/L spectinomycin or supplemented with various concentrations of theophylline. wt plants grown on medium with 17 mg/L spectinomycin served as phenocopy controls. For light treatments, plants were grown under standard conditions in a walk-in chamber (25°C, humidity 55%, 16 h light/8 dark). Homoplastomy of the regenerates and seedlings of T1 generation were tested by Southern Blot Analysis and/or germination assay on a medium containing 500 mg/L spectinomycin.

### Construction of the Synthetic *matK* (AmatK) Overexpression Vector

The tobacco plastid rRNA operon promoter Prrn and riboswitch RS were amplified from plasmid pAV6 (Verhounig et al., 2010) with primer pair SacIf 5′-GAACAAAAGCTGGAGCTCG-3′ (SacI site underlined) and AmatKRSr 5′-GTAACGCTGGATTTCTTCCATATGATCCTCTCCACGAGAG-3′ (sequence overlap with AmatK underlined). The PCR product was ligated to the AmatK fragment via overlap PCR with primer pair SacIf and HA5AmatKr 5′-CATAATCAGGAACATCATAAGGATACTGGTAGTTTGCCAGGTC-3′ (sequence overlap with HA underlined). The resulting PCR product was ligated to the 3XHA tag (Zoschke et al., 2010) via overlap PCR with primer pair SacIf and pAVHAr 5′-CCTTAATTGAATTTCTCTAGAGCCTCATTAAGCATAATCAGGTACATC-3′ (XbaI site underlined). The resulting PCR product and pAV6 vector were digested with SacI and XbaI and ligated, resulting in the pRSAmatK vector.

### Plastid Transformation

Stable transformation of plastids was performed by particle bombardment followed by spectinomycin selection. The transformation protocol was carried out according to the protocols of Svab (Svab and Maliga, 1993) and Okuzaki (Okuzaki and Tabei, 2012). Two rounds of transformation were carried out; in the first round, two transplastomic lines with variegated cotyledons were retrieved, in the second round more than a dozen independent transplastomic lines with cotyledon variegation were isolated. Briefly, sterile tobacco leaves from wild type plants cultured on MS were cut into 0.5 cm × 0.5 cm squares. The leaf pieces were placed onto MS plates with the lower leaf side up and cultured overnight. The particle bombardment was carried out using the Biolistic PDS-1000/He Particle Delivery System (1,100 psi, L2 = 6 cm, 10 shots per construct, Bio-Rad) with gold particles (0.6 μM, Bio-Rad) loaded with the transformation plasmids. Spectinomycin resistant calli were regenerated on RMOP medium. Several putative transplastomic lines were obtained and further rounds of selection were carried out to generate homoplastomic lines. The correct integration of AmatK was verified by genotyping PCR and Southern blot. The PCR was performed with the primer pair AmatKseqf 5′-GTTCTCGCATTTGGATCTC-3′ and trnMf 5′-TTCAAATCCTGTCTCCGCAA-3′.

### Isolation of DNA and Southern Blot

Genomic DNA was isolated from leaves according to standard protocols (Murray and Thompson, 1980). For Southern blot analysis, *Hind*III-digested total plant DNA was separated on a 0.8% agarose gel, blotted to a Nylon membrane and probed with a PCR product that was body-labeled using α-^32^P-CTP. The Decaprime DNA labeling Kit (ThermoFisher, Berlin, Germany) was used to label PCR-generated probe DNA fragments (see **Supplementary Table S1** for primers). Hybridization was carried out at 55°C overnight using standard protocols (Sambrook et al., 1989).

### Isolation of RNA and RNA Gel Blot Hybridization

Total cellular RNA was extracted using TriZol (ThermoFisher, Berlin, Germany). 5 μg of total cellular RNA was separated on formaldehyde-containing 1.2% agarose gels and blotted to Hybond N membrane (GE healthcare, Munich, Germany). Strand-specific RNA probes were synthesized using T7 Polymerase (ThermoFisher, Berlin, Germany) with radioactive γ-^32^P-UTP (Hartmann Analytic) using PCR products as templates (see **Supplementary Table S1** for primer sequences). Strand-specific DNA probes were generated with the DecaLabel kit and PCR products according to the manufacturer’s instructions (ThermoFisher, Berlin, Germany; see **Supplementary Table S1** for primer sequences). Alternatively, oligonucleotides were end-labeled using T4 polynucleotide kinase (ThermoFisher, Berlin, Germany) and γ-^32^P-UTP (Hartmann Analytic) and used as probes (see **Supplementary Table S1** for oligonucleotide sequences). Hybridizations were done according to standard procedures (Sambrook et al., 1989). Signals were detected using a PMI Imaging system (Bio-Rad, Munich, Germany).

### Immunoblot Analyses

Protein extraction was performed according to standard protocols (Schagger and von Jagow, 1987). Protein fractions were separated on polyacrylamide gels and subsequently transferred in Towbin buffer (Towbin et al., 1979) to a PVDF membrane (0.2 μm pore size; GE Healthcare, Munich, Germany). Integrity and equal loading of proteins was detected by Ponceau S staining of the membrane. MatK:HA detection was performed with a rat HA-specific antibody (Roche, Mannheim, Germany). Chemoluminescent signals were detected using a ChemiDoc system (BioRad, Munich, Germany).

### Microarray Analysis

2 μg of total cellular RNA from 10 day old AmatK and phenocopy control plants with red- (Cy5) and green-fluorescing (Cy3) dyes, respectively, using the ULS RNA Fluorescent Labeling Kit according to the manufacturer’s instructions (Kreatech, Amsterdam, Netherlands). The samples were hybridized to a whole-genome tobacco chloroplast tiling microarrays (Finster et al., 2013). Hybridization, array washings and signal detection were carried out as described previously (Kupsch et al., 2012).

Microarray analysis was done in *R* using the packages *marray* (version 1.52) and *multtest* (version 2.28). Namely, expression values were loaded in R using *marray*. After this, loess normalization at the probe level was applied using *maNorm* function from the *marray* package for the two biological replicates performed. The array contains 12 copies of each probe located at different spots to reduce any bias regarding its location on the array; the median log2 fold-change expression for the 12 copies was used to calculate the log2 fold-change of the region represented by the probe for each biological replicate independently. A *t*-test was applied to calculate which regions show an average log2 fold-change different of zero. Multiple hypotheses correction was applied using the Benjamini & Hochberg method (BH) implemented in the *multitest* package.

## Author Contributions

YQ designed the transformation vectors, performed chloroplast transformation and selection for homoplastomic lines, and performed genetic crosses. JA and AW analyzed transgene expression and splicing efficiency by RNA gel blot hybridization. JL performed genotyping PCRs and Southern analyses. SH and GH performed RNA gel blot analyses and microarray analyses. CT performed immunological analyses. GR participated in the screening for transplastomic plants. JM and UO evaluated the microarrays and contributed to manuscript preparation. GW supported the design of the transformation vector and immunological analyses. OO contributed to project design and manuscript preparation. CS-L conceived the study, prepared the figures, and wrote the manuscript.

## Conflict of Interest Statement

The authors declare that the research was conducted in the absence of any commercial or financial relationships that could be construed as a potential conflict of interest.
