# Supplementary material for: Ectopic Transplastomic Expression of a Synthetic MatK Gene Leads to Cotyledon-Specific Leaf Variegation
Source: Front Plant Sci. 2018 Oct 4;9:1453. doi: 10.3389/fpls.2018.01453 (PMC6180158; doi:10.3389/fpls.2018.01453)
Supplement: Supplementary file 1 [file Data_Sheet_1.PDF]

```
NTmatK     ATGGAAGAAATCCAAAGATATTTACAGCCAGATAGATCGCAACAACACAACTTCCTATAT
AmatK      ATGGAAGAAATCCAGCGTTACCTGCAACCGGATCGCTCCCAGCAACACAATTTCCTCTAC
           **************  * **  * ** ** *** * ** ** ******** ***** **
           MetGluGluIleGlnArgTyrLeuGlnProAspArgSerGlnGlnHisAsnPheLeuTyr

NTmatK     CCACTTATCTTTCAGGAGTATATTTATGCACTTGCTCATGATCATGGTTTAAATAGAAAT
AmatK      CCGCTGATCTTCCAAGAGTACATTTATGCGCTCGCTCACGACCACGGTCTGAACCGCAAC
           ** ** ***** ** ***** ******** ** ***** ** ** *** * **  * **
           ProLeuIlePheGlnGluTyrIleTyrAlaLeuAlaHisAspHisGlyLeuAsnArgAsn

NTmatK     AGGTCGATTTTGTTGGAAAATCCAGGTTATAACAATAAATTAAGTTTCCTAATTGTGAAA
AmatK      CGTAGCATTCTCCTCGAAAATCCGGGCTACAATAACAAGCTGTCTTTCCTGATTGTAAAA
            *    *** *  * ******** ** ** ** ** **  *   ****** ***** ***
           ArgSerIleLeuLeuGluAsnProGlyTyrAsnAsnLysLeuSerPheLeuIleValLys

NTmatK     CGTTTAATTACTCGAATGTATCAACAGAATCATTTTCTTATTTCTACTAATGATTCTAAC
AmatK      CGCCTGATCACGCGTATGTACCAACAGAATCACTTTCTCATTTCTACCAACGACTCTAAC
           **  * ** ** ** ***** *********** ***** ******** ** ** ******
           ArgLeuIleThrArgMetTyrGlnGlnAsnHisPheLeuIleSerThrAsnAspSerAsn

NTmatK     AAAAATTCATTTTTGGGGTGCAACAAGAGTTTGTATTCTCAAATGATATCAGAGGGATTT
AmatK      AAGAACAGCTTTCTCGGTTGTAACAAATCTCTGTACTCCCAGATGATTTCTGAAGGTTTC
           ** **    *** * ** ** *****   * **** ** ** ***** ** ** ** **
           LysAsnSerPheLeuGlyCysAsnLysSerLeuTyrSerGlnMetIleSerGluGlyPhe

NTmatK     GCGTTTATTGTGGAAATTCCGTTTTCTCTACGATTAATATCTTCTTTATCTTCTTTCGAA
AmatK      GCGTTCATCGTAGAGATCCCATTTTCCCTCCGCCTCATCTCTTCTCTGTCTAGCTTCGAG
           ***** ** ** ** ** ** ***** ** **  * ** ****** * ***   *****
           AlaPheIleValGluIleProPheSerLeuArgLeuIleSerSerLeuSerSerPheGlu

NTmatK     GGCAAAAAGATTTTTAAATCTTATAATTTACGATCAATTCATTCAACATTTCCTTTTTTA
AmatK      GGTAAGAAAATCTTCAAGTCTTATAACCTGCGCTCTATCCATTCCACGTTCCCTTTTCTC
           ** ** ** ** ** ** ********  * ** ** ** ***** ** ** ****** *
           GlyLysLysIlePheLysSerTyrAsnLeuArgSerIleHisSerThrPheProPheLeu

NTmatK     GAGGACAATTTTTCACATCTAAATTATGTATTAGATATACTAATACCCTACCCTGTTCAT
AmatK      GAAGATAATTTTTCCCACCTGAATTACGTCCTGGATATTCTCATCCCTTACCCGGTTCAC
           ** ** ******** ** ** ***** **  * ***** ** ** ** ***** *****
           GluAspAsnPheSerHisLeuAsnTyrValLeuAspIleLeuIleProTyrProValHis

NTmatK     CTGGAAATCTTGGTTCAAACTCTTCGCTATTGGGTAAAAGATGCCTCTTCTTTACATTTA
AmatK      CTGGAAATCCTGGTTCAAACGCTCCGCTACTGGGTAAAGGACGCCAGCAGCCTGCACCTG
           ********* ********** ** ***** ******** ** ***       * **  *
           LeuGluIleLeuValGlnThrLeuArgTyrTrpValLysAspAlaSerSerLeuHisLeu

NTmatK     TTACGATTCTTTCTCCATGAATTTTGGAATTTGAATAGTCTTATTACTTCAAAGAAGCCC
AmatK      CTGCGCTTCTTCCTCCACGAGTTCTGGAATCTCAACAGCCTCATTACCTCTAAGAAACCA
            * ** ***** ***** ** ** ****** * ** ** ** ***** ** ***** **
           LeuArgPhePheLeuHisGluPheTrpAsnLeuAsnSerLeuIleThrSerLysLysPro

NTmatK     GGTTACTCCTTTTCAAAAAAAAATCAAAGATTCTTCTTCTTCTTATATAATTCTTATGTA
AmatK      GGCTATTCCTTCTCCAAGAAGAACCAACGCTTTTTTTTCTTTCTGTACAACAGCTATGTC
           ** ** ***** ** ** ** ** *** * ** ** *****  * ** **    *****
           GlyTyrSerPheSerLysLysAsnGlnArgPhePhePhePheLeuTyrAsnSerTyrVal

NTmatK     TATGAATGCGAATCCACTTTCGTCTTTCTACGGAACCAATCTTCTCATTTACGATCAACA
AmatK      TATGAATGCGAATCTACCTTTGTTTTCCTGCGTAACCAATCTTCCCATCTGCGTAGCACC
           ************** ** ** ** ** ** ** *********** *** * **    **
           TyrGluCysGluSerThrPheValPheLeuArgAsnGlnSerSerHisLeuArgSerThr

NTmatK     TCTTTTGGAGCCCTTCTTGAACGAATATATTTCTATGGAAAAATAGAACGTCTTGTAGAA
```

```
AmatK      TCTTTCGGTGCGCTCCTGGAGCGTATCTACTTCTACGGCAAAATCGAACGCCTCGTTGAA
           ***** ** ** ** ** ** ** ** ** ***** ** ***** ***** ** ** ***
           SerPheGlyAlaLeuLeuGluArgIleTyrPheTyrGlyLysIleGluArgLeuValGlu

NTmatK     GTCTTTGCTAAGGATTTTCAGGTTACCCTATGGTTATTCAAGGATCCTTTCATGCATTAT
AmatK      GTTTTCGCAAAGGACTTTCAAGTGACGCTGTGGCTGTTCAAAGACCCGTTCATGCACTAC
           ** ** ** ***** ***** ** ** ** *** * ***** ** ** ******** **
           ValPheAlaLysAspPheGlnValThrLeuTrpLeuPheLysAspProPheMetHisTyr

NTmatK     GTTAGGTATCAAGGAAAATCCATTCTGGCTTCAAAAGGGACGTTTCTTTTGATGAATAAA
AmatK      GTTCGTTACCAGGGTAAATCTATTCTGGCAAGCAAAGGCACCTTCCTGCTCATGAACAAA
           *** * ** ** ** ***** ********    ***** ** ** **  * ***** ***
           ValArgTyrGlnGlyLysSerIleLeuAlaSerLysGlyThrPheLeuLeuMetAsnLys

NTmatK     TGGAAATTTTACCTTGTCAATTTTTGGCAATGTCATTGTTCTCTGTGCTTTCACACAGGA
AmatK      TGGAAGTTCTATCTGGTGAACTTTTGGCAGTGCCATTGCTCTCTGTGCTTCCACACAGGT
           ***** ** ** ** ** ** ******** ** ***** *********** ********
           TrpLysPheTyrLeuValAsnPheTrpGlnCysHisCysSerLeuCysPheHisThrGly

NTmatK     AGGATCCATATAAACCAATTATCCAATCATTCCCGTGACTTTATGGGCTATCTTTCAAGT
AmatK      CGTATTCATATCAACCAACTGTCCAACCACTCTCGTGACTTTATGGGTTATCTGAGCAGC
            * ** ***** ****** * ***** ** ** ************** *****   **
           ArgIleHisIleAsnGlnLeuSerAsnHisSerArgAspPheMetGlyTyrLeuSerSer

NTmatK     GTGCGACTAAATCCTTCAATGGTACGTAGTCAAATGTTAGAAAATTCATTTCTAATCAAT
AmatK      GTACGTCTGAACCCGTCTATGGTTCGTAGCCAGATGCTGGAGAACTCCTTTCTGATCAAC
           ** ** ** ** ** ** ***** ***** ** *** * ** ** ** ***** *****
           ValArgLeuAsnProSerMetValArgSerGlnMetLeuGluAsnSerPheLeuIleAsn

NTmatK     AATGCAATTAAGAAGTTCGATACCCTTGTTCCAATTATTCCTTTGATTGGATCATTAGCT
AmatK      AACGCGATCAAAAAGTTTGACACGCTGGTTCCGATCATTCCGCTCATCGGCTCCCTGGCG
           ** ** ** ** ***** ** ** ** ***** ** *****  * ** ** **  * **
           AsnAlaIleLysLysPheAspThrLeuValProIleIleProLeuIleGlySerLeuAla

NTmatK     AAAGCAAACTTTTGTACCGTATTAGGGCATCCCATTAGTAAACCGGTTTGGTCCGATTTA
AmatK      AAGGCGAATTTCTGCACCGTCCTCGGTCATCCAATTTCTAAGCCAGTTTGGTCTGACCTG
           ** ** ** ** ** *****  * ** ***** ***  *** ** ******** **  *
           LysAlaAsnPheCysThrValLeuGlyHisProIleSerLysProValTrpSerAspLeu

NTmatK     TCAGATTCTGATATTATTGACCGATTTGGGCGTATATGCAGAAATCTTTTTCATTATTAT
AmatK      TCTGACTCTGACATCATCGACCGTTTCGGTCGTATCTGCCGTAATCTGTTTCACTACTAC
           ** ** ***** ** ** ***** ** ** ***** *** * ***** ***** ** **
           SerAspSerAspIleIleAspArgPheGlyArgIleCysArgAsnLeuPheHisTyrTyr

NTmatK     AGCGGATCTTCCAAAAAAAAGACTTTATATCGAATAAAGTATATACTTCGACTTTCTTGT
AmatK      TCTGGTTCTAGCAAAAAGAAAACCCTCTATCGCATCAAGTACATCCTGCGCCTCTCTTGT
              ** ***  ****** ** **  * ***** ** ***** ** ** ** ** ******
           SerGlySerSerLysLysLysThrLeuTyrArgIleLysTyrIleLeuArgLeuSerCys

NTmatK     GCTAGAACTTTAGCTCGGAAACACAAAAGTACTGTACGCACTTTTTTGAAAAGATCGGGC
AmatK      GCCCGCACGCTCGCGCGTAAACACAAAAGCACGGTTCGCACTTTCCTCAAACGTAGCGGC
           **  * **  * ** ** *********** ** ** ********  * *** *    ***
           AlaArgThrLeuAlaArgLysHisLysSerThrValArgThrPheLeuLysArgSerGly

NTmatK     TCGGAATTATTGGAAGAATTCTTAACGTCGGAAGAACAAGTTCTTTCTTTGACCTTCCCA
AmatK      TCTGAGCTGCTGGAAGAGTTTCTGACCAGCGAAGAGCAGGTACTGTCCCTGACTTTTCCG
           ** **  *  ******* **  * **    ***** ** ** ** **  **** ** **
           SerGluLeuLeuGluGluPheLeuThrSerGluGluGlnValLeuSerLeuThrPhePro
```

```
NTmatK     CGAGCTTCTTCTAGTTTGTGGGGAGTATATAGAAGTCGGATTTGGTATTTGGATATTTTT
AmatK      CGTGCATCCTCTAGCCTGTGGGGCGTTTATCGTTCTCGCATTTGGTATCTCGACATCTTC
           ** ** ** *****  ******* ** *** *   *** ********* * ** ** **
           ArgAlaSerSerSerLeuTrpGlyValTyrArgSerArgIleTrpTyrLeuAspIlePhe

NTmatK     TGTATCAATGATCTGGCGAATTATCAA
AmatK      TGTATCAACGACCTGGCAAACTACCAG
           ******** ** ***** ** ** **
           CysIleAsnAspLeuAlaAsnTyrGln
```

Suppl. Fig. 1: Alignment of wild type *matK* (NTmatK) and the mutated version of *matK* used in this study (AmatK). Asterisks denote identical nucleotides (72% of all nucleotides are identical between AmatK and NTmatK). Codons changed between NTmatK and AmatK are underlined.
