# Supplementary material for: Ectopic Transplastomic Expression of a Synthetic MatK Gene Leads to Cotyledon-Specific Leaf Variegation
Source: Front Plant Sci. 2018 Oct 4;9:1453. doi: 10.3389/fpls.2018.01453 (PMC6180158; doi:10.3389/fpls.2018.01453)
Supplement: Supplementary file 2 [file Data_Sheet_2.PDF]

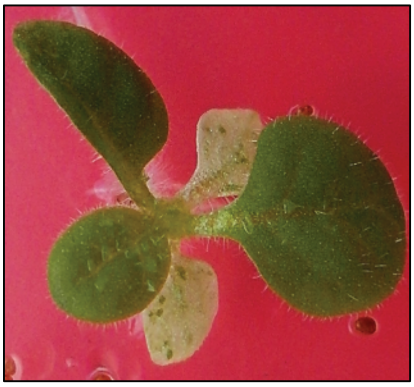

Supplemental Figure 2: Phenotype ofAmatK plant after 18 days of growth on MS medium. Note that while cotely-dons show strong bleaching, primary leaves are green.
