# Supplementary material for: Ectopic Transplastomic Expression of a Synthetic MatK Gene Leads to Cotyledon-Specific Leaf Variegation
Source: Front Plant Sci. 2018 Oct 4;9:1453. doi: 10.3389/fpls.2018.01453 (PMC6180158; doi:10.3389/fpls.2018.01453)
Supplement: Supplementary file 3 [file Data_Sheet_3.PDF]

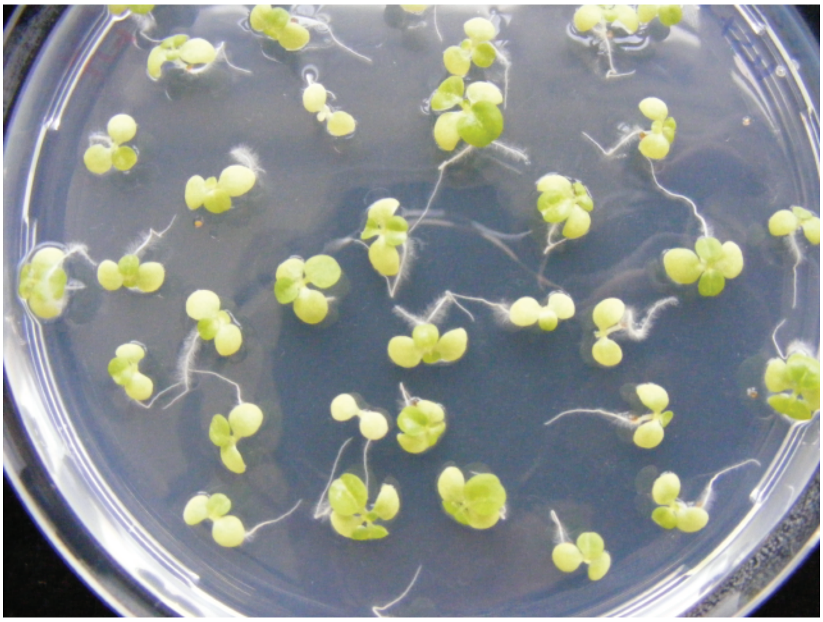

Supplemental Figure 3: Phenotype of wt plants grown on MS medium containing 17 µg / L spectinomycin. These plants are used as phenocpy controls since they have pale cotelydons, while the primary leaves are green.
