# Supplementary material for: Ectopic Transplastomic Expression of a Synthetic MatK Gene Leads to Cotyledon-Specific Leaf Variegation
Source: Front Plant Sci. 2018 Oct 4;9:1453. doi: 10.3389/fpls.2018.01453 (PMC6180158; doi:10.3389/fpls.2018.01453)
Supplement: Supplementary file 4 [file Data_Sheet_4.PDF]

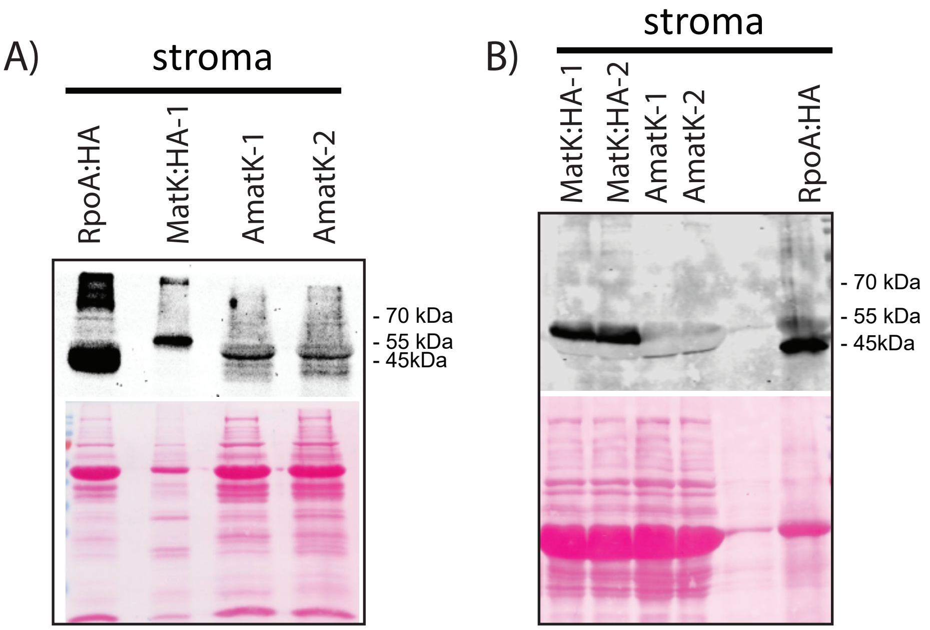

Supplemental Figure 4: Immunological analysis of MatK:HA in AmatK lines. In these experiments, a signal distinct in size from MatK:HA control lines can be seen slightly above 45 kDA (as in the blot shown in Fig. 4). This is a replicate of the experiment shown in figure 4, with the following differences: A) An anti-HA antibody from mouse was used (Sigma-Aldrich) for detection B) nitrocellulose (Whatman NC20, GE Healthcare, Chalfont St. Giles, GB) was used as a blotting membrane and 0.1% SDS was added to the transfer buffer.The IRDye 800CW goat anti mouse antibody (Li-Cor, Lincoln, USA) was used as a secondary antibody. Detection was done with Odyssey CLx Imaging system (LI-Cor, Lincoln, USA).
