# Supplementary material for: Ectopic Transplastomic Expression of a Synthetic MatK Gene Leads to Cotyledon-Specific Leaf Variegation
Source: Front Plant Sci. 2018 Oct 4;9:1453. doi: 10.3389/fpls.2018.01453 (PMC6180158; doi:10.3389/fpls.2018.01453)
Supplement: Supplementary file 5 [file Data_Sheet_5.PDF]

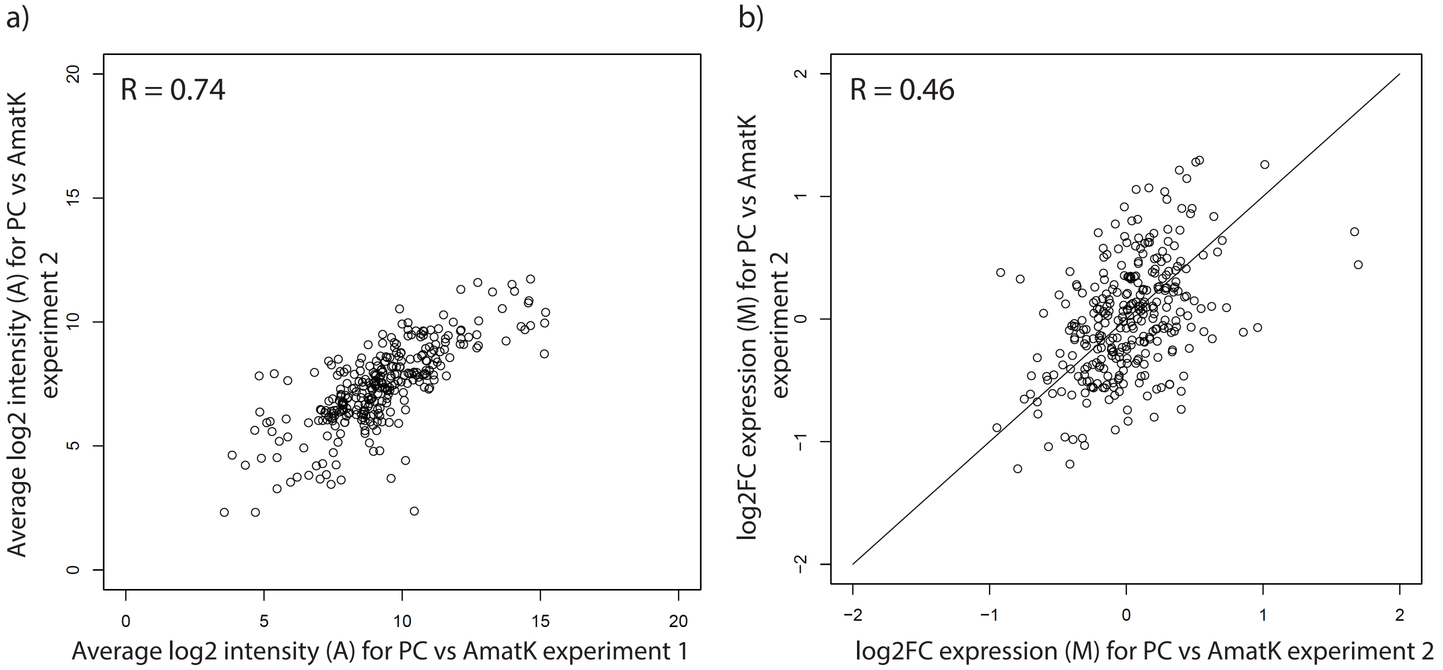

Supplemental Figure 5: Reproducibility of the analysis. a) The average log2 signal intensity from the combined green and red channel signals for each region represented on the array from experiment 2 are plotted against the corresponding values from experiment 1. The Pearson correlation coefficient of 0.74 indicates the high reproducibility of the analysis. b) The difference between log2 signal intensities of the green versus red channel are plotted for each biological replicate; replicate 1 on the x-axis and replicate 2 on the y-axis. The Pearson correlation coeffcient is low, further corroborating that there is no signifcant change in RNA accumulation between the two tissue types. PC = phenocopy (seedlings grown on low levels of spectinomycin to induce cotyledon bleaching).
