# Supplementary material for: Ectopic Transplastomic Expression of a Synthetic MatK Gene Leads to Cotyledon-Specific Leaf Variegation
Source: Front Plant Sci. 2018 Oct 4;9:1453. doi: 10.3389/fpls.2018.01453 (PMC6180158; doi:10.3389/fpls.2018.01453)
Supplement: Supplementary file 6 [file Data_Sheet_6.PDF]

Supplemental Table 1: microarray data for Fig. 6a

Name	log2FC[1]	Standard Deviation[2]	Pvalue[3]	BH[4]
trnH	0.224559	0.779962271	0.753838	0.860726
psbA-3'	-0.40886	0.202940654	0.214886	0.661839
psbA-1	-0.0475	0.089013891	0.588413	0.794001
psbA-2	-0.17999	0.23124056	0.469493	0.72558
psbA-3	0.006065	0.069678029	0.92203	0.956133
psbA-trnK	-0.10615	0.047303574	0.194341	0.661839
matK-3'	-0.18504	0.082807929	0.195101	0.661839
matK-1	0.005111	0.032425124	0.860361	0.936905
matK-2	-0.11814	0.267740791	0.644832	0.82907
matK-3	-0.35127	0.05723372	0.073024	0.572955
matK-4	-0.17097	0.261302266	0.524676	0.733132
matK-5'UTR	-0.0429	0.416264793	0.907866	0.951394
trnK-5'-1	-0.24	0.006406636	0.012015	0.572364
trnK-5'-2	-0.28217	0.087249936	0.137036	0.66131
rps16-3'	0.151629	0.576064052	0.773139	0.876225
rps16-Ex2-In	-0.27516	0.025996446	0.042466	0.572364
rps16-In	-0.31339	0.178385953	0.243608	0.661839
rps16-5'	0.006643	0.189435397	0.968456	0.981283
Ig-trnQ-3'	0.041208	0.383499272	0.903993	0.951394
trnQ-psbK	0.163716	0.043060084	0.117061	0.66131
trnQ-psbK-psbI	-0.36395	0.367823796	0.395009	0.71314
psbK-psbI-trnS	0.1994	0.161016726	0.330289	0.700363
Ig-trnG-5'UTR	0.057018	0.018663177	0.144797	0.66131
trnG-Ex1-In	-0.22611	0.237430513	0.406606	0.71314
trnG-In	-0.09945	0.133331534	0.483016	0.726896
trnG-In-Ex2	-0.23235	0.107924133	0.202027	0.661839
trnG-trnR	-0.43411	0.052683732	0.054498	0.572364
trnR-atpA	-0.1922	0.061055648	0.140662	0.66131
atpA-1	0.308816	0.300452385	0.383625	0.71314
atpA-2	0.287315	0.133797393	0.202511	0.661839
atpA-atpF	0.236414	0.552715872	0.653668	0.829342
atpF-Ex2	0.179348	0.09815422	0.235064	0.661839
atpF-Ex2-In	0.106029	0.598830816	0.843802	0.922155
atpF-In-Ex1	-0.1109	0.49363569	0.804153	0.898069
atpF-In	-0.04847	0.207359198	0.796766	0.894403
atpF-atpH	-0.14887	0.061039859	0.179651	0.661839
atpH	0.180568	0.183320529	0.396378	0.71314
Ig-atpH-atpI	-0.02319	0.286730797	0.927488	0.956133
atpI	0.358491	0.285494005	0.326499	0.700363
atpI-rps2-3'UTR	0.492306	0.581143805	0.442799	0.71314
rps2-1	0.1616	0.420367097	0.682987	0.829342
rps2-2	0.405712	0.210574307	0.223927	0.661839
rps2-rpoC2	0.118865	0.352285284	0.716565	0.84988
rpoC2-1	0.398394	0.157375738	0.173404	0.661839
rpoC2-2	0.131174	0.142393152	0.41677	0.71314
rpoC2-3	-0.10832	0.225459991	0.620055	0.810841
rpoC2-4	0.221062	0.281368706	0.466528	0.72558

rpoC2-5	0.093672	0.700382158	0.880994	0.947493
rpoC2-6	0.368246	0.221496187	0.25601	0.661839
rpoC2-rpoC1	-0.44574	0.727795225	0.545591	0.758867
rpoC1-Ex2-In	-0.02659	0.002195177	0.037119	0.572364
rpoC1-In	0.536931	0.055083617	0.046101	0.572364
rpoC1-In-Ex1-1	-0.06737	0.20902196	0.727719	0.856469
rpoC1-In-Ex1-2	0.174126	0.559700524	0.736133	0.859519
rpoC1-rpoB-1	0.266226	0.301946846	0.430322	0.71314
rpoC1-rpoB-2	-0.0342	0.425884412	0.928011	0.956133
rpoB-1	0.156773	0.231031533	0.513105	0.73028
rpoB-2	0.409099	0.081738575	0.089351	0.601377
rpoB-3	0.572932	0.048503243	0.038064	0.572364
rpoB-5'	0.450968	0.211999921	0.204304	0.661839
Ig-rpoB-trnC-1	0.353288	0.378551235	0.41278	0.71314
Ig-rpoB-trnC-2	-1.19174	0.675889427	0.242804	0.661839
trnC-petN	-0.67074	0.042690625	0.028632	0.572364
petN	-1.13762	0.175381301	0.069126	0.572364
petN-psbM	-0.41307	0.451638115	0.418986	0.71314
psbM	0.167752	0.221678748	0.478424	0.726896
psbM-5'UTR	-0.42132	0.538300737	0.467734	0.72558
trnD-3'UTR	-0.33161	0.484940297	0.510658	0.73028
trnD-trnY-trnE-1	-0.19266	0.474242786	0.668015	0.829342
trnD-trnY-trnE-2	-0.33146	0.374044332	0.428762	0.71314
trnY-trnE-trnT	-0.73897	0.139980418	0.084767	0.601377
trnE-trnT	-0.32686	0.291195088	0.357878	0.71314
trnT-5'UTR	-0.91513	0.538332606	0.250949	0.661839
trnT-psbC	-0.6536	0.351357078	0.231255	0.661839
psbD-5'UTR	0.040922	0.197123762	0.818206	0.903866
psbD-1	0.200833	0.231076343	0.434793	0.71314
psbD-2	0.181635	0.009124613	0.022605	0.572364
psbD-3	0.390962	0.375914201	0.380127	0.71314
psbD-4	0.126671	0.03955014	0.138332	0.66131
psbD-psbC	0.159043	0.154976479	0.384087	0.71314
psbC-1	0.06425	0.151246474	0.6556	0.829342
psbC-2	0.012714	0.091293096	0.876202	0.947413
psbC-3	-0.12637	0.31767494	0.673778	0.829342
psbC-trnS	-0.39649	0.142102704	0.15801	0.661839
trnS-orf105	-0.51876	0.303638087	0.249817	0.661839
orf105-psbZ	-0.15188	0.305140694	0.609529	0.810229
psbZ-trnG-trnfM	-0.80123	0.584990746	0.3034	0.697905
trnfM-rps14	-0.41909	0.349677434	0.339338	0.701604
rps14-psaB-3'UTR	0.254507	0.28910917	0.430811	0.71314
rps14-psaB	0.284073	0.169230694	0.253811	0.661839
psaB-1	0.295891	0.136653553	0.200949	0.661839
psaB-2	-0.13736	0.20636168	0.519237	0.733132
psaB-3	0.276077	0.307203606	0.424408	0.71314
psaB-4	0.197625	0.172057305	0.351306	0.711918
psaB-psaA	0.400077	0.21744176	0.233582	0.661839
psaA-1	0.338059	0.278435722	0.335736	0.701604
psaA-2	-0.42397	0.019306154	0.020491	0.572364

psaA-3	0.16801	0.803616112	0.816987	0.903866
psaA-5'	0.121411	0.177693786	0.510917	0.73028
Ig-psaA-ycf3	-0.19025	0.22446677	0.442643	0.71314
ycf3-EX3-In2	-0.25877	0.446237002	0.562726	0.772171
ycf3-In2	-0.10457	0.382432953	0.76509	0.870326
ycf3-In2-Ex2	-0.27023	0.258616338	0.378738	0.71314
ycf3-In1	-0.34925	0.203167846	0.24844	0.661839
ycf3-Ex2-In-Ex1	-0.39767	0.322098489	0.331127	0.700363
ycf3-Ex1-orf74	-0.16991	0.4744095	0.701529	0.841041
orf74-trnS(GGA)	-0.03017	0.248966457	0.891956	0.947703
trnS(GGA)-rps4	-0.30976	0.066615785	0.096073	0.604481
rps4-trnT(UGU)	-0.44817	0.516087375	0.435053	0.71314
trnT(UGU)-orf70A	-0.34736	0.604586164	0.565612	0.772666
orf70A-trnL	-0.10053	0.028440057	0.125697	0.66131
trnL-1	0.034731	0.263015043	0.882469	0.947493
trnL-2	-0.33278	0.040963034	0.055273	0.572364
trnL-In	-0.23845	0.297800713	0.460529	0.722996
Ig-trnL-ndhJ	-0.36272	0.195174095	0.231457	0.661839
ndhJ	-0.36186	0.150367872	0.18194	0.661839
ndhJ-ndhK	-0.29125	0.117693309	0.177186	0.661839
ndhK-ndhC	-0.3749	0.404306807	0.414757	0.71314
ndhC-5'UTR	-0.25416	0.016141589	0.02857	0.572364
trnV(UAC)-3'UTR	-0.32689	0.047574816	0.065285	0.572364
trnV(UAC)-In	-0.24676	0.02123846	0.038697	0.572364
trnV(UAC)-trnM(CAU)	-0.55864	0.236025399	0.184817	0.661839
trnV-trnM-atpE-3'UTR	-0.47567	0.175349651	0.162331	0.661839
trnM(CAU)-atpE	0.159861	0.400991471	0.673176	0.829342
atpE	0.663699	0.016390032	0.011116	0.572364
atpE-atpB	0.48706	0.279166707	0.245136	0.661839
atpB-1	0.509122	0.157344164	0.136968	0.66131
atpB-2	0.53265	0.082545157	0.069484	0.572364
atpB-3	0.14885	0.040903157	0.122179	0.66131
atpB-5'UTR	-0.0855	0.010696835	0.056171	0.572364
atpB-rbcL-5'UTR	-0.02023	0.063583579	0.730865	0.856876
rbcL-1	0.179851	0.406972108	0.644395	0.82907
rbcL-2	0.200574	0.135436596	0.28359	0.694351
rbcL-3	0.037437	0.052859144	0.499492	0.73028
rbcL-4	0.255138	0.101947742	0.175304	0.661839
rbcL-5	0.415659	0.129536368	0.138081	0.66131
rbcL-3'UTR	0.087814	0.174856147	0.606852	0.810229
accD-5'	-0.89498	0.546639161	0.259545	0.661839
accD	-0.5637	0.69691578	0.457337	0.722996
accD-3'-orf71A	-0.66523	0.276337908	0.181882	0.661839
orf71A-psaI	-0.6603	0.536273955	0.331871	0.700363
psaI-ycf4	-0.08546	0.028888483	0.149368	0.661839
ycf4-ycf10	-0.10903	0.142404455	0.474704	0.726548
ycf10-1	-0.26783	0.02536912	0.042575	0.572364
ycf10-2	-0.25819	0.034647577	0.060228	0.572364
ycf10-petA	-0.37965	0.027665608	0.032775	0.572364
petA	-0.12226	0.003406498	0.012541	0.572364

petA-3'UTR-orf99	-0.54241	0.027556424	0.02286	0.572364
orf99-psbJ-psbL	-0.07657	0.201515427	0.686078	0.829802
psbL-psbF-psbE	-0.30329	0.174788452	0.24635	0.661839
psbE	-0.32367	0.245483174	0.313385	0.697905
psbE-orf103	-0.18047	0.207522577	0.434603	0.71314
orf103-5'	-0.08911	0.056517123	0.268387	0.673167
Ig-orf103-petL	-0.20371	0.382468587	0.589014	0.794001
petL-petG-trnW(CCA)	-0.30164	0.067118347	0.09935	0.608023
petG-trnW(CCA)-trnP(U	0.226436	0.245049955	0.415827	0.71314
trnP(UGG)-psaJ-1	0.208003	0.035686275	0.076856	0.58795
trnP(UGG)-psaJ-2	0.524686	0.08050264	0.068799	0.572364
psaJ-rpl33	0.91656	0.042124586	0.020682	0.572364
rpl33-rps18	0.655535	0.062847293	0.043092	0.572364
rps18-rpl20	0.323526	0.326913338	0.394958	0.71314
rpl20-5'UTR	0.337596	0.073156851	0.096796	0.604481
rps12-3'UTR	0.119109	0.292965248	0.667805	0.829342
clpP-In2	0.029779	0.135137795	0.80768	0.898728
rps12-clpP-In2	0.41052	0.598109744	0.509477	0.73028
clpP-In2-clpP-Ex2	0.364751	0.274482839	0.311311	0.697905
clpP-Ex2-In1	0.297799	0.709309019	0.658893	0.829342
clpP-In1	-0.12287	0.113169053	0.36751	0.71314
clpP-In1-Ex1-1	-0.17478	0.267134553	0.524692	0.733132
clpP-In1-Ex1-2	0.068854	0.296433886	0.797948	0.894403
psbB-1	-0.10065	0.248907937	0.669288	0.829342
psbB-2	0.129156	0.296061202	0.648085	0.829342
psbB-psbT-1	-0.30974	0.139183441	0.195861	0.661839
psbB-psbT-2	-0.4505	0.351507776	0.320964	0.697905
psbH	-0.30398	0.106242476	0.154241	0.661839
psbT-psbN-psbH	-0.18039	0.058167725	0.142715	0.66131
psbH-petB-Ex1-In-1	-0.3923	0.330071403	0.341666	0.701676
psbH-petB-Ex1-In-2	-1.07061	0.88602256	0.337065	0.701604
petB-In	-0.12148	0.035283188	0.128957	0.66131
petB-In-Ex2	-0.17715	0.236145975	0.481186	0.726896
petB-Ex2	-0.37699	0.341014308	0.362272	0.71314
petB-petD-Ex1-In	-0.20173	0.204019804	0.395217	0.71314
petD-In	-0.33989	0.33451899	0.387056	0.71314
petD-In-Ex2	-0.09541	0.262748791	0.697974	0.840866
petD-Ex2	-0.15001	0.068897334	0.199914	0.661839
petD-Ex2-rpoA-5'UTR	-0.28706	0.138464333	0.20926	0.661839
petD-Ex2-rpoA	-0.21055	0.19997448	0.376504	0.71314
rpoA	-0.00258	0.041352705	0.943937	0.969278
rpoA-rps11	0.111945	0.156522789	0.496378	0.73028
rps11-rpl36	0.274946	0.359283067	0.474868	0.726548
rpl36-5'	0.228157	0.08922513	0.171751	0.661839
rpl36-5'UTR-rps8	0.227718	0.023529968	0.046432	0.572364
rps8-rpl14	-0.06803	0.057930067	0.345051	0.703904
rpl14-rpl16-Ex2	-0.14488	0.075688319	0.225276	0.661839
rpl16-Ex2-In	-0.08191	0.058572844	0.298032	0.697905
rpl16-In-1	-0.0168	0.201701045	0.925344	0.956133
rpl16-In-2	0.109403	0.060547046	0.237468	0.661839

rpl16-In-Ex1-rps3	-0.17815	0.082583012	0.201647	0.661839
rps3	-0.04198	0.056562215	0.484597	0.726896
rps3-rpl22	0.095053	0.200547143	0.624071	0.81262
rpl22-rps19	0.035719	0.033963885	0.376834	0.71314
rpl22-rps19-rpl2-Ex2	-0.13679	0.004324869	0.014231	0.572364
rps19-rpl2-Ex2	-0.19527	0.14110778	0.300731	0.697905
rpl2-Ex2-In	0.321702	0.383119423	0.445565	0.713837
rpl2-In-Ex1	-0.07931	0.059673484	0.311274	0.697905
rpl2-In	0.018977	0.174107426	0.902638	0.951394
rpl2-Ex2-rpl23	-0.29765	0.047194315	0.071078	0.572364
rpl23-trnI(CAU)	-0.45047	0.656516381	0.509577	0.73028
trnI(CAU)-ycf2	-0.79434	0.498391937	0.265832	0.67227
ycf2-1	0.268766	0.918916039	0.750316	0.860726
ycf2-2	-0.60745	0.084199634	0.062198	0.572364
ycf2-3	-0.07142	0.242826726	0.74905	0.860726
ycf2-4	-0.01443	0.347369013	0.962641	0.981283
ycf2-5	0.189196	0.147962025	0.321583	0.697905
ycf2-6	0.047057	0.443737688	0.905231	0.951394
ycf2-7	0.054465	0.140084048	0.679953	0.829342
ycf2-8	0.176061	0.032175302	0.081813	0.601377
ycf2-9	0.099159	0.151518175	0.524614	0.733132
ycf2-10	0.38211	0.14442675	0.166261	0.661839
ycf2-ycf15	0.278344	0.461704406	0.550554	0.760071
ycf15-orf92-orf115	0.482007	0.237892213	0.21376	0.661839
orf92-orf115-trnL	-0.61705	0.640800911	0.403231	0.71314
trnL-orf79-ndhB-Ex2	0.254069	0.230455366	0.363062	0.71314
ndhB-Ex2	0.578253	0.163566775	0.125675	0.66131
ndhB-Ex2-In	0.147408	0.488567888	0.743251	0.860726
ndhB-In	-0.04677	0.094735746	0.611946	0.810229
ndhB-In-Ex1	0.178959	0.120865805	0.28364	0.694351
ndhB-Ex1	0.406331	0.001323084	0.001466	0.448532
ndhB-Ex1-rps7	0.685924	0.417505022	0.258744	0.661839
rps7-1	1.006582	0.301821749	0.133009	0.66131
rps7-2	0.668106	0.512320611	0.316309	0.697905
rps7-rps12	0.797213	0.543964013	0.286183	0.695015
rps12	0.706115	0.363078315	0.222007	0.661839
rps12-Ex1-5'UTR	0.804482	0.33372673	0.181646	0.661839
Ig-rps12-orf131-orf70b	0.309121	0.087764717	0.126131	0.66131
orf131-trnV(GAC)	0.028065	0.226598149	0.889613	0.947703
trnV(GAC)-rrn16	0.052397	0.117355587	0.641452	0.82907
rrn16-1	-0.27823	0.269317154	0.382108	0.71314
rrn16-2	-0.18786	0.217486844	0.436713	0.71314
rrn16-3	0.064428	0.209412895	0.738737	0.859519
rrn16-trnI-Ex1-1	0.27222	0.148547952	0.234441	0.661839
trnI(GAU)-1	-0.23218	0.472612919	0.613441	0.810229
trnI(GAU)-2	-0.07366	0.214264375	0.711917	0.847652
trnI(GAU)-3	-0.37422	0.680659504	0.579267	0.787803
trnI(GAU)-trnA(UGC)	-0.23428	0.556370502	0.658061	0.829342
trnA(UGC)-Ex1-In	0.146513	0.304286106	0.619416	0.810841
trnA(UGC)-In	0.027337	0.21913876	0.88883	0.947703

trnA(UGC)-In-Ex2	0.022662		0.626602547	0.967467	0.981283
trnA(UGC)-rrn23-2	-0.3157		0.325671815	0.401208	0.71314
rrn23-1	0.019933		0.001298296	0.0293	0.572364
rrn23-2	-0.31365		0.43777078	0.495813	0.73028
rrn23-3	-0.09976		0.141579779	0.501115	0.73028
rrn23-4	-0.04143		0.046435458	0.426642	0.71314
rrn23-5	-0.20468		0.52837757	0.680943	0.829342
rrn23-6	-0.08107		0.340360715	0.793153	0.894403
rrn23-rrn4.5-1	-0.04189		0.052352499	0.460733	0.722996
rrn23-rrn4.5-2	-0.19066		0.14853537	0.320542	0.697905
rrn23-rrn4.5-3	-0.01012		0.224425189	0.959455	0.981283
rrn23-rrn4.5-rrn5	-0.10503		0.004183414	0.017925	0.572364
rrn5-trnR(ACG)	0.054263		0.376718247	0.872068	0.946286
trnR(ACG)-trnN(GUU)-o	-0.17989		0.218099483	0.451177	0.719063
trnN(GUU)-orf350	-0.10982		0.30912667	0.703616	0.841041
ndhF-3'-1	0.176637		0.259040739	0.511556	0.73028
ndhF-3'-2	0.345761		0.068121944	0.088123	0.601377
ndhF-1	0.365694		0.530030707	0.507818	0.73028
ndhF-2	-0.00476		0.174379013	0.975436	0.985094
ndhF-3	0.15974		0.030538898	0.085542	0.601377
ndhF-rpl32-1	0.002752		0.240758957	0.989711	0.989711
ndhF-rpl32-2	0.219817		0.226326702	0.400626	0.71314
rpl32	0.22582		0.155537347	0.288528	0.695193
ccsA-5'UTR	-0.24962		0.644217274	0.680874	0.829342
rpl32-sprA/fs24	0.052725		0.162994124	0.726861	0.856469
trnL-ccsA-1	-0.63621		0.480125185	0.312063	0.697905
trnL-ccsA-2	-0.69228		0.298559423	0.188436	0.661839
ccsA-1	0.057052		0.196407784	0.75186	0.860726
ccsA-2	0.17145		0.088671979	0.223198	0.661839
ccsA-ndhD	0.201852		0.05800614	0.127624	0.66131
ndhD-1	0.215166		0.213599749	0.389637	0.71314
ndhD-2	0.183923		0.12198336	0.279171	0.694351
ndhD-psaC-2	0.380271		0.105630317	0.123471	0.66131
psaC-ndhE	0.356444		0.07365673	0.092368	0.601377
ndhE-ndhG-1	0.399905		0.226724062	0.242726	0.661839
ndhE-ndhG-2	0.492452		0.05496109	0.050137	0.572364
ndhG	0.710686		0.089993461	0.056851	0.572364
ndhG-ndhI	0.300276		0.232610551	0.319026	0.697905
ndhI	0.698337		0.066770632	0.042976	0.572364
ndhI-ndhA-1	0.419125		0.064964883	0.069498	0.572364
ndhI-ndhA-2	0.053131		0.263776325	0.823332	0.906258
ndhA-Ex2	0.094066		0.088335337	0.373171	0.71314
ndhA-Ex2-In	0.01263		0.56477401	0.979873	0.986319
ndhA-In	0.287413		0.097027007	0.149176	0.661839
ndhA-In-Ex1-1	0.387754		0.165246805	0.186332	0.661839
ndhA-In-Ex1-2	0.132503		0.102557897	0.318801	0.697905
ndhA-In-Ex1-3	-0.09532		0.01037492	0.048902	0.572364
ndhA-In-Ex1	0.207606		0.242991724	0.440136	0.71314
ndhA-Ex1-ndhH	0.254412		0.05162714	0.09073	0.601377
ndhH-1	0.265439		0.158473468	0.254305	0.661839

ndhH-2	0.253954	0.073972236	0.129314	0.66131
ndhH-rps15	0.213144	0.435099583	0.614292	0.810229
rps15-ycf1-1	0.108715	0.601724769	0.840746	0.922108
ycf1	-0.00421	0.23780057	0.984079	0.987305
rpl2-trnH	-0.4697	0.233066868	0.214827	0.661839

[1] Average log2 expression foldchange of F532 (AmatK) over F635 (PC) across two biological replicate experiments

[2] Standard deviation of the log2 expression foldchange between biological replicates (with each 12 technical replicates; i.e. signals for in total 24 DNA spots for each probe)

[3] p-value obtained after applying a t-test

[4] adjusted p-value after applying a multiple hypotheses correction using the Benjamini & Hochberg method
